# Correlation of Cardiac Output by Arterial Contour-Derived Cardiac Output Monitor Versus Pulmonary Artery Catheter in Liver Transplant: Experience at an Indian Center

**DOI:** 10.5152/TJAR.2021.1356

**Published:** 2022-04-01

**Authors:** Kusuma Halemani, Lakshmi Kumar, Bhadrinath Narayanan, Sunil Rajan, Pavithra Ramamurthi, Abish Sudhakar

**Affiliations:** 1 Department of Anaesthesia, Amrita Institute of Medical Sciences, Kochi, Kerala, India; 2 Department of Pediatric Cardiology, Amrita Institute of Medical Sciences, Kochi, Kerala, India

**Keywords:** Arterial pulse derived cardiac output monitor, cardiac output, FloTrac, liver transplantation, pulmonary artery catheter

## Abstract

**Objective:**

Arterial pulse-derived cardiac output monitors are routinely employed to guide hemodynamic management during liver transplant surgery. In this study, we sought to assess the reliability by evaluating the agreement of the cardiac output measured by the FloTrac Vigileo versus pulmonary artery catheter (continuous cardiac output) at specified times during liver transplant.

**Methods:**

Liver transplant database with cardiac output values measured by FloTrac Vigileo and continuous cardiac output was analyzed retrospectively at a tertiary care hospital. Data were compared at T0: baseline, T1: 1 hour in dissection phase, T2: anhepatic phase, T3: portosystemic shunt, T4: reperfusion, T5: 1 hour after reperfusion, and T6: skin closure. Statistical analysis was done using Bland–Altman analysis and percentage error (<30%) to assess the agreement between cardiac output measured by 2 techniques, Lin’s concordance correlation coefficient for quantifying the agreement and 4-quadrant plots to compare the trends of cardiac output.

**Results:**

Bland–Altman analysis showed mean cardiac output ± standard deviation L min^-1^ (95% CI) at T0: 0.2 ± 2.09 (−3.9 to 4.3), T1: 0.53 ± 3.0 (−5.4 to 6.4), T2: 0.47 ± 2.1(−3.7 to 4.6), T3: 0.31 ± 1.9 (−3.4 to 4.0), T4: 0.44 ± 2.15 (−3.8 to 4.7), T 5:0.69 ± 1.9. (−2.9 to 4.3), and at T6: 0.43 ± 2.25 (−4.0 to 4.8). Percentage error was 44%-72% and concordance correlation coefficient was poor (<0.65) at all points.

**Conclusions:**

There is poor agreement between the cardiac output measured by FloTrac and pulmonary artery catheter among liver transplant recipients. The need for superior hemodynamic monitoring is mandated in liver transplant.

**Main Points**

- FloTrac, although simple, is not as reliable as continuous cardiac output during liver transplant surgeries.
- Even though intermittent boluses measuring cardiac output and transesophageal echocardiogram (TEE) are superior, these techniques have their own limitations and complications in cirrhotic patients undergoing liver transplant surgery.
- The reliability of thermodilution technique used by pulmonary artery catheter for measuring cardiac output is questionable in the presence of large temperature shifts that occur during liver transplant.
- Recent literature reviews suggest that TEE appears to be more informative, reliable, and safe if used following proper guidelines even in the presence of varices.

## Introduction

Liver transplantation surgery is associated with major hemodynamic shifts associated with blood losses in the background of an altered cardiovascular state. Cardiovascular complications are the major cause for post-transplant mortality^1^ and among them, perioperative cardiac dysfunction is commonly known to occur.^2^ Traditionally, Swan–Ganz catheter has been used to monitor cardiac output (CO); however, its use is diminishing on account of its invasiveness and limited utility. The transesophageal echocardiogram (TEE) is a reliable alternative; however, its availability, lack of technical expertise, and potential bleeding from esophageal varices have curtailed its use in cirrhotic liver disease (CLD) patients. 

Minimally invasive CO monitors such as the FloTrac Vigileo™ (FTCO) derive the CO from arterial contour incorporating heart rate (HR), patient demographics, and aortic compliance over a period of time. Its advantages are in its apparent simplicity and lack of need for calibration.^3,4^ However, a poor agreement in comparison to continuous cardiac output (CCO) monitoring in patients undergoing liver transplant surgeries, critically ill, or patients on high-dose vasoconstrictors has been reported.^5^ Despite these observations, minimally invasive monitors are extensively used in many centers to guide hemodynamic management during transplant surgery.

In this retrospective analysis, we primarily sought to compare the reliability of FloTrac by evaluating the agreement between CO measured from a third-generation FTCO with that derived from CCO from a Swan–Ganz pulmonary artery catheter (PAC) at specified time points during surgery. Third-generation FloTrac software (version 3.02) database, unlike the previous versions, relies on a larger dataset^6^ to calculate the proprietary correction factor “khi’ that automatically adjusts for changes in vascular tone, so that a wider range of peripheral resistances can be accommodated (dynamic tone technology). This has been shown to give better overall precision and trending ability in comparison to earlier versions.^7^

## Methods

**Figure 1.**
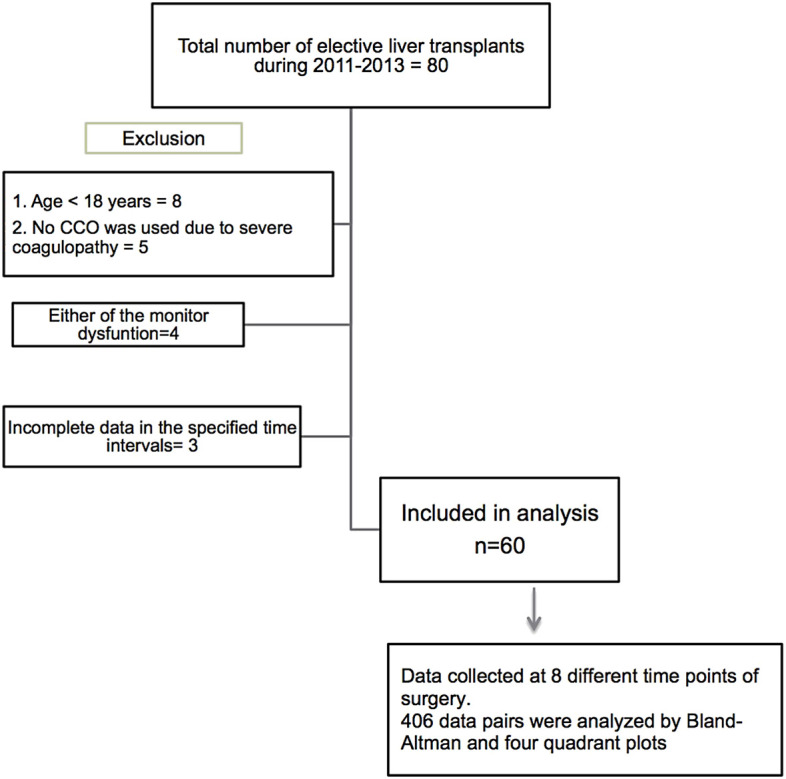
Flow diagram of selection of patients.

Following ethical committee approval (IEC-AIMS/2020/ANES-052), a retrospective analysis of data records was performed in 60 patients who underwent elective living donor transplant surgery at our institution. As FloTrac was newly introduced in our institution at the time point in the study, all patients had simultaneous measurements of CO with FloTrac and Swan–Ganz CCO monitor for intraoperative monitoring. The patient management was based on CCO findings. The data were obtained from case file archives and from electronic medical records at our hospital (Figure 1).

In all the cases, anasthesia was administered as per standard protocols. Radial artery was cannulated under local anaesthesia prior to induction of anaesthesia. Induction was accomplished with intravenous lorazepam 1-2 mg, fentanyl 2 μg kg^-1^, and propofol titrated to the loss of verbal response. Succinylcholine 1.5 mg kg^-1^ was used for intubation and subsequent neuromuscular blockade maintained with atracurium infusion 0.005-0.01 mg kg^-1^ min^-1^. Anaesthesia was maintained with 50 : 50 air oxygen mixtures at 1.0 L flow and isoflurane at minimum alveolar concentration between 0.7 and 1.0. Radial arterial line was connected through FloTrac sensor to the output monitor, (FloTrac Vigileo™, Edward Life Sciences, Irvine, Calif, USA) third-generation device. Right internal jugular vein was cannulated and a 9 French triple lumen catheter with sheath (Edwards Life Sciences) was inserted. Swan–Ganz, PAC with CCO monitoring, (Edwards Life Sciences LLC), was then inserted and positioning was confirmed with the trace.

Comparisons of CO were performed as per protocol at predefined time points. First sets of readings of CCO and simultaneous FTCO were taken when the thermo-dilution monitor was connected to the patient as baseline (T0). Readings between both methods of CO determination were compared at specific time points during the surgery that included T1, 1 hour into dissection, the start of anhepatic T2, at the time of portal shunt T3, at reperfusion T4, 1 hour after reperfusion T5, and at skin closure T6. Monitoring as per protocol was discontinued at the end of surgery and patients were shifted to the intensive care unit for management. All adult transplants with complete data and with the use of both methods of CO monitoring were included for analysis.

Sample size was estimated by comparing CO at reperfusion (T4) between PAC and FloTrac (time at which greatest difference is expected) among the first 10 patients in the study group. Mean CO was determined as 6.87 ±1.1 L min^-1^ versus 7.68 ± 2.13 L min^-1^ between the PAC and FloTrac, respectively. With 90% power and 95% CI, the minimum sample size was calculated as 44. We included 60 patients in our study for agreement analysis. 

Bias, precision, percentage error, and limits of agreement (LOA) between CO by both methods were calculated in accordance with Bland–Altman. Bias was calculated as the mean difference between CO measured by both methods. Precision represents the random error or variability in agreement between the 2 techniques and was calculated as the standard deviation (SD) of the difference between CO by both methods. The LOA was calculated as “bias ± (1.96* SD)”as suggested by Bland–Altman.^8^

Percentage error was calculated as per formula, “(1.96* SD of bias)/mean CO” and value of <30% was considered as significant in establishing agreement as suggested by Critchley et al.^9^ Four-quadrant plots with 0.5 L min^-1^ exclusion zone were plotted to compare the trends in CO with measured time points during surgery.^10^ Lin’s concordance correlation coefficient (CCC) that quantifies the agreement between 2 measures of CO was calculated; value less than <0.9 was considered as poor agreement, values 0.90-0.95 as moderate, 0.95-0.99 as substantial, and >0.99 as perfect agreement.^11^ Statistical analysis was done using IBM Statistical Package for the Social Sciences 20.0 (IBM Corp.; Armonk, NY, USA) and MedCalc version 19.4.

## Results

Data from 60 patients were obtained and included for analysis. Demographic data are included in Table 1. Mean duration of surgery was 9.47 ± 1.60 hours. A total of 406 data pairs distributed over 7 different time points were collected. 

**Figure 2.**
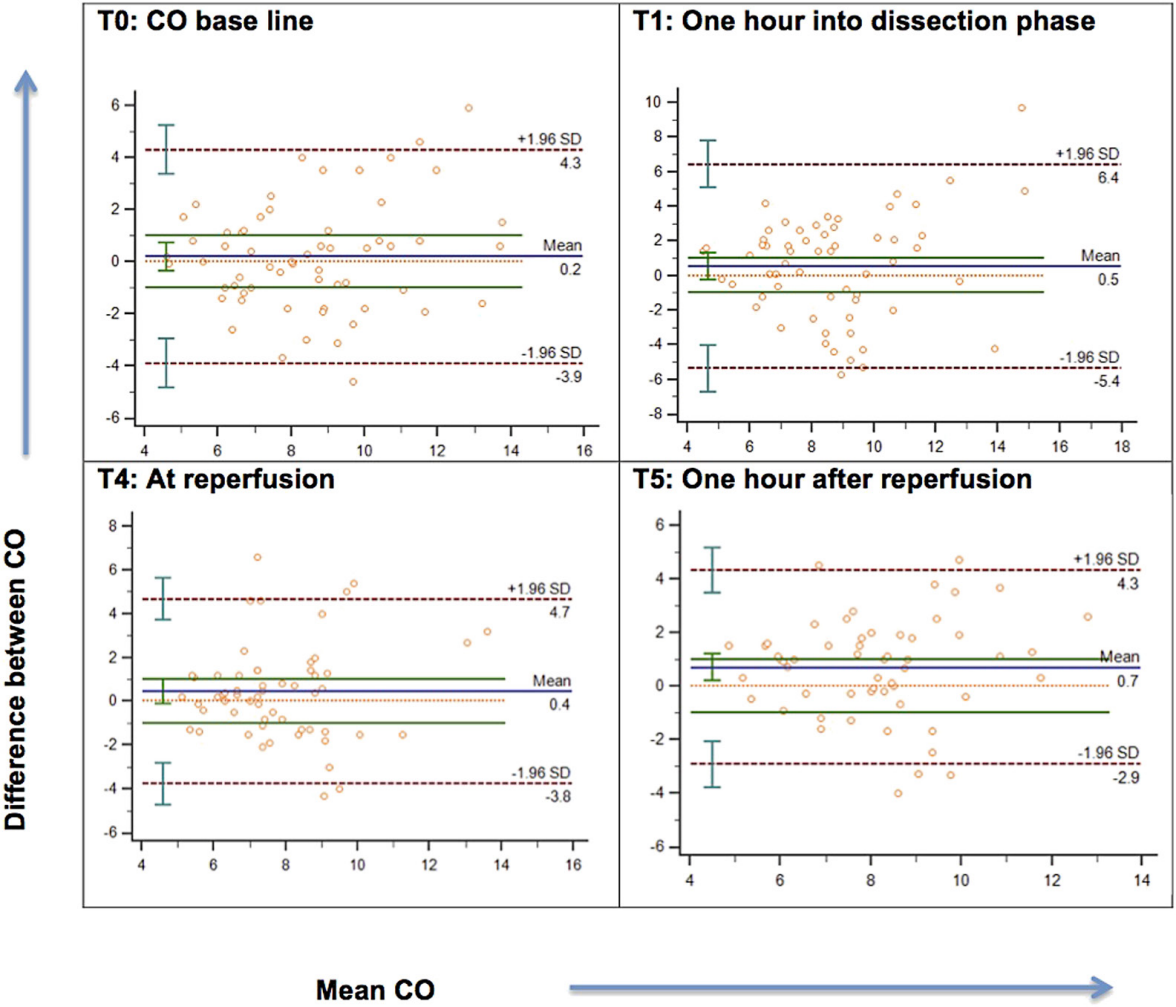
Bland–Altman graphs comparing CO from CCO and FloTrac. CO, cardiac output; CCO, continuous cardiac output.

The CO measured by both methods was compared by Band–Altman method (Table 2, Figure 2) At the start of the surgery T0, the bias was 0.2 L, precision was 2.09 L, 95% CI was −3.9 to 4.3, with 61.03% error. At T3, time of shunt, the bias was 0.31 L, precision was 1.9 L, and 95% CI was −3.4 to 4.0, with 46.91% error. At T4, reperfusion, the bias was 0.44 L, precision was 2.15 L, and 95% CI was −3.8 to 4.7, with 54.15% error. At T6, time of skin closure, the bias was 0.43 L, precision was 2.25 L, and 95% CI was −4.0 to 4.9 with an error of 72.34%. The LOA and 95% CIs for LOA were well above the set limit of an acceptable limit of 1 L min^-1^ (Figure 2). The percentage error was >30% at all the time points (Table 2). 

**Figure 3.**
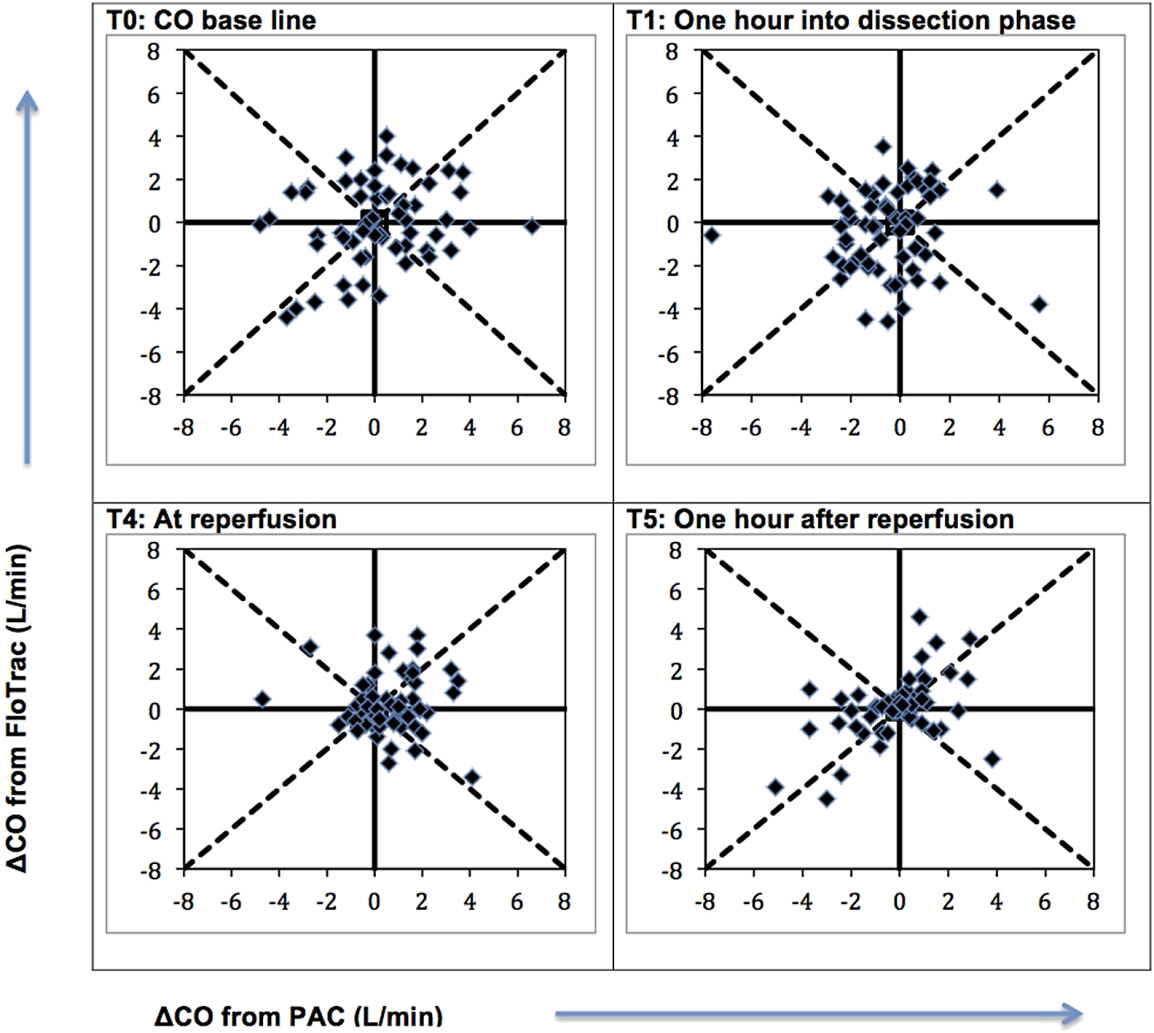
Four quadrant plots comparing CO from CCO and FloTrac. CO, cardiac output; CCO, continuous cardiac output.

Four-quadrant plots with 0.5 L min^-1^ exclusion zone, plotted to compare the trends in CO with measured time points during surgery, showed that the trends in CO were not similar between the 2 methods of measurement (Figure 3). Lin’s CCC was applied to quantify the degree of correlation, which was below 0.9 at all time points during comparison implying a poor agreement between the 2 methods (Table 2).

The central venous pressure, stroke volume variation (SVV), HR, Mean Arterial Pressure (MAP), and systemic vascular resistance (SVR) were also measured during the study and are summarized in Table 3.

## Discussion

We compared the CO measured by Swan–Ganz CCO with a newer minimally invasive device FloTrac™, with the aim of evaluating agreement between the 2 methods. Bland–Altman analysis revealed poor agreement between the 2 methods (% error > 45%), and 4-quadrant plots showed that the directions of change between the 2 methods were dissimilar at all points included for the study.

Eventhough PAC is the standard method of measurement of CO, there are reports on its limitation for its use in liver transplant recipients. Other than bleeding complications associated with PAC, the incidence of ventricular arrhythmia during PA catheter insertion in liver transplant recipients is found to be higher^12^ than critically ill patients (37% vs 12.7%). Underlying cardiomyopathy, prolonged QTc interval, and prolonged transit time in the right ventricle due to its enlargement might be the reasons.^12^ Among the recipients in our study, none had documented sustained arrhythmias during the insertion of the PAC or during the perioperative period.

FloTrac Vigileo monitor was introduced as a simpler, minimally invasive method in the measurement of CO and it has been used extensively in non-cardiac surgeries including liver transplant. The arterial pulse is sampled at 20-second intervals and stroke volume inferred from a constant quantifies arterial resistance and compliance.^13^ The accuracy of CO is based upon the integrity of the arterial waveform that can be altered in the presence of peripheral arteriolar vasoconstriction.^14^ However, Lee et al.^15^ while comparing FTCO from radial and femoral arteries have concluded that the caliber of the artery did not affect the measurements. A number of studies among cardiac surgical patients have documented good agreement between FloTrac and PAC.^16,17^

We used Bland–Altman analysis to compare the agreement between CO measured by 2 methods. The LOA showed maximal variation between −5.4 L min^-1^ and +6.4 L min^-1^ at T1, which was 1 hour in the dissection phase. This meant that the FTCO could range from 5.4 L min^-1^ less than or 6.4 L min^-1^ more than the CCO, which is well above the acceptable difference of 1.0 L min^-1^ raising concerns on its validity to guide clinical decisions.

Further analyses of data pictorially by 4-quadrant plots for the trending of CO showed that the data were randomly distributed and did not trend in the same direction. Lin’s CCC was calculated to quantify the results and we found poor agreement (<0.9) between the techniques at all the time points. 

Similar to other studies in liver transplant surgeries,^7,18,19^ we did not find good agreement between FTCO and CCO. Third-generation FloTrac has been shown to be more precise than earlier versions,^7^ but we encountered a percentage error of >30% when compared to CCO among our patients. FloTrac-derived CO appears to correlate with that derived from the stat mode thermodilution in Child A, B and C are grades derived from Child-Pugh grading system for cirrhotic liver disease. Child A: 5-6 points, Child B: 7-9 points and Child C: 10-15 points. The scoring system is based on encephalopathy, ascites, serum bilirubin levels, Prothrombin time/ international normalized ratio (INR) and serum albumin levels.^13^ As patients presenting for transplant are likely to be in child B or C, and 54% of our patients were child C, the poor agreement seems explainable in the group of patients studied.

Biais^13^ and Rocca et al.^20^ have observed that the bias and 95% LOA between CCO and arterial pulse CO (APCO) increased significantly in hyperdynamic conditions with CO > 8.0 L min^-1^. Our patients had CO higher than 8.0 L min^-1^. The reliability of FloTrac in low SVR states^5^ and in the background use of the high vasopressor therapy is poor^21^ and this is similar to the clinical profile during transplant surgery.

Another explanation for the fallibility of the FloTrac in the setting of CLD patients is that extreme splanchnic dilatation results in an uneven distribution of the blood volume and APCO monitors function on an assumption of evenly distributed CO^18^ This would imply that CO as assessed by a peripheral artery waveform cannot reflect global CO changes in comparison to the thermodilution.

Pulmonary artery catheter-based monitoring is informative by directly measuring and calculating data, which are required for proper patient care perioperatively. However, its accuracy may decrease when thermal noise increases due to rapid changes in CO occur such as when caval clamping is required or when peripheral intravenous fluid infusion rates are high.^13^ Intermittent boluses measuring CO (ICO), although alluded to as the clinical gold standard, is known to have errors relating to the speed of injection, phase of the respiratory cycle, use of volume resuscitation at the time of measurement, and presence of tricuspid and mitral regurgitant valvular lesions.^22^

In terms of the frequency of measurements, FloTrac measures CO every 20 seconds while CCO at best would measure these at intervals of 90-120 seconds. It is possible that FloTrac may be a more sensitive monitor of sudden hemodynamic changes during the transplant surgery than CCO.^23^

Stroke volume variation measured by FTCO is a reliable predictor of fluid requirement in transplant surgery.^24^ Kim et al.^25^ have demonstrated a good correlation between SVV and right ventricular end-diastolic index (RVEDI) measured by PAC in liver transplant surgery. We did not record the RVEDI during the surgery.

The need for an accurate CO monitor in high-risk non-cardiac surgery is imminent and a balance of availability, costs, need for technical expertise, and safety from adverse side effects need to be considered. Availability of smartphone-based applications widens the horizons for the assessment of CO based upon dynamic parameters with minimal costs.^26^

Transesophageal echocardiogram is now being routinely used in high volume liver transplant centers, and the presence of gastro-esophageal varices is considered as a relative contraindication.^27,28^ Markin et al.^29^ suggested proper preoperative screening, insertion of probe by an experienced person, limited probe manipulation, and avoiding transgastric view as guidelines for the prevention of bleeding complications (0.86%). De Pietri et al.^27^ and guidelines by the American association of liver disease, however, suggest that the presence of esophageal varices is not a contraindication for the use of TEE if the indications are justifiable.^30^

Direct visualization of the heart in real-time in TEE allows for instantaneous assessment of changes in global and regional contractility, rapid diagnosis of ventricular dilatation and failure, rapid optimization of hypovolemia, intraoperative diagnosis, and management of porto-pulmonary hypertension, air embolism, and thromboembolism.^27^ Transesophageal echocardiogram can overcome the limitations of PAC measurements arising from large core body temperature shifts due to massive fluid infusion or revascularization of the new graft during liver transplant.^28^

We acknowledge the limitations as ours was a retrospective study, we did not have gold standard ICO monitor, the data points were measured at the specified time of recording, and we did not correlate the measurements with ongoing blood loss or volume replacement. However, we wish to highlight the limitations of the third generation FloTrac in liver transplant and endorse the need for alternate perioperative monitoring. Pulmonary artery catheter has been considered as the reference standard and TEE is emerging as an alternative and both are associated with potential risks with their use.

## Conclusion

There is poor agreement between cardiac output measured by FloTrac and pulmonary artery catheter during liver transplant surgery. This mandates the need for a more reliable and relatively safe hemodynamic monitor during transplant surgeries.

## Figures and Tables

**Table 1.** Demographics of Patients

Variable	Mean ± SD
Age (years)	46.62 ± 8.47
Height (cm)	167.8 ±7.27
Weight (kg)	73.24 ± 12.15
MELD score	23.2 ± 5.56
Male: female (n)	54 : 6
Child–Pugh score “C” (n(%))	54 (90%)

MELD, model for end-stage liver disease; SD, standard deviation.

**Table 2.** Bland–Altman Analysis of Cardiac Output and Concordance Correlation Coefficient

Time Points	n	Bias (L min^-1^)	SD/Precision	95% CI of the Difference (L min^-1^)		% Error (1.96^*^SD Bias/Mean CO)		Concordance Correlation Coefficient
				Lower Limit	Upper Limit			
T0	60	0.2	2.09	−3.9	4.3	61.03	0.65	
T1	60	0.53	3	−5.4	6.4	68.08	0.39	
T2	60	0.47	2.1	−3.7	4.6	50.28	0.59	
T3	59	0.31	1.9	−3.4	4.0	46.91	0.63	
T4	59	0.44	2.15	−3.8	4.7	54.15	0.43	
T5	56	0.69	1.9	−2.9	4.3	44.67	0.52	

T0, Baseline; T1, 1 hour in dissection phase; T2, anhepatic phase; T3, portosystemic shunt; T4, reperfusion; T5, 1 hour after reperfusion; T6, skin closure.

**Table 3.** Hemodynamic Parameters Measured from CCO and FloTrac

Parameter	T0	T1	T2	T3	T4	T5	T6	Overall Mean ± SD
Mean CO by CCO (L min^-1^)	8.61 ± 2.7	8.93 ± 2.9	8.46 ± 2.6	8.1 ± 2.4	8.01 ± 2.1	8.46 ± 2.0	8.37 ± 1.9	8.42 ± 2.41
Mean CO by FloTrac (L min^-1^)	8.41 ± 2.3	8.41 ± 2.5	8.0 ± 2.19	7.79 ± 2.1	7.57 ± 1.9	7.77 ± 1.9	7.94 ± 2.3	7.98 ± 2.20
SVR by CCO (dynes s cm^-5^)	672.1 ± 243	658.7 ± 252	635.9 ± 175	647.5 ± 174	666 ± 190	660.5 ± 183	621 ± 157	653.7 ± 200
SVR by FloTrac (dynes s cm^-5^)	646.5 ± 196	674 ± 229	643.8 ± 168	662 ± 187	674.9 ± 192	675.7 ± 187	614.3 ± 167	660.4 ± 192
CVP (mmHg)	14.33 ± 4.2	11.71 ± 4	11.65 ± 3.8	12.18 ± 4.7	11.62 ± 4.9	12.44 ± 4.4	12.67 ± 5.5	12.2 ± 4.6
SVV	6.42 ± 2.6	7.07 ± 3	6.85 ± 2.8	6.56 ± 3.3	6.91 ± 3.7	6.51 ± 3.57	6.16 ± 3.1	6.6 ± 3.2
Heart Rate (beats min^-1^)	85.4 ± 8.4	85.3 ± 8.5	95.14 ± 8.7	99.1 ± 7.8	87.3 ± 10.8	86.9 ± 9.3	87.7 ± 10.5	89.5 ± 5.4
MBP (mm Hg)	79 ± 12	80 ± 13.9	75.4 ± 10.1	73.1 ± 12.2	75.6 ± 9.7	77 ± 9.9	76.3 ± 11.5	76.6 ± 11.5
T0, Baseline; T1, 1 hour in dissection phase; T2, anhepatic phase; T3, portosystemic shunt; T4, reperfusion; T5, 1 hour after reperfusion; T6, skin closure; CO, cardiac output; CCO, continuous cardiac output; SVR, systemic vascular resistance; CVP, central venous pressure; SVV, stroke volume variation; MBP, mean blood pressure.								
